# 1491. *Life's Simple 7 (LS7)* is Not So Simple in Newly Diagnosed People Living with HIV: Evaluating Associations Between HIV and LS7 in ART-Naive People Living with HIV in Tanzania

**DOI:** 10.1093/ofid/ofad500.1326

**Published:** 2023-11-27

**Authors:** Safah Khan, Gloria J Manyangu, Robert N Peck, George Praygod

**Affiliations:** Weill Cornell Medicine-Qatar, Maryland; Bugando Medical Centre, Mwanza, Tanzania, Mwanza, Mwanza, Tanzania; Weill Cornell Medical College, New York City, New York, USA, New York, New York; National Institute of Medical Research, Dar Es Salaam, Tanzania, Dar Es Salam, Dar es Salaam, Tanzania

## Abstract

**Background:**

People living with HIV (PLWH) have complex cardiovascular health (CVH) profiles due to a combination of traditional, HIV-specific and lifestyle factors. Thorough characterization is necessary to guide primary prevention efforts. Previous studies that evaluated CVH in PLWH were mainly done in those on stable antiretroviral therapy (ART) and data in newly diagnosed PLWH is scarce. Therefore, we applied *Life's Simple 7 (LS7)* to compare CVH in ART-Naive PLWH and HIV-uninfected adults in Tanzania.

**Methods:**

A cross-sectional analysis was conducted on a cohort of ART-naive PLWH and HIV-uninfected adults recruited from HIV clinics in Mwanza, Tanzania (CICADA cohort). We applied modified *Life's Simple Seven (LS7)* definitions (**Table 1)** and compared the distribution of LS7 metrics between study groups. Each LS7 was dichotomized as "ideal" (1 point) and "not ideal" (0 points) to obtain a total LS7 score. Poisson regressions were employed to investigate associations between HIV status and count of ideal total LS7 components (0-7). Logistic regression was employed to investigate individual LS7 metrics. Regressions were adjusted for age, sex, education, income.

**Figure:**
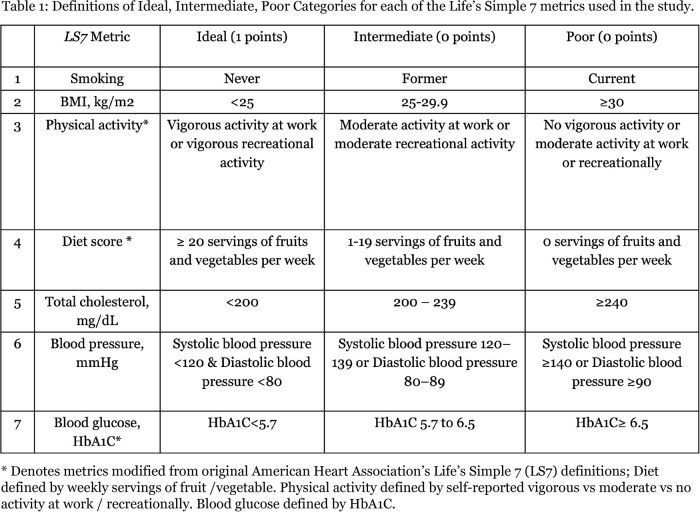

**Results:**

Our study included 1,224 participants (860 PLWH, 364 controls). PLWH had higher total LS7 scores (4.16 vs 3.85, p=0.015) and higher prevalence of ideal overall CVH (40% vs 35%, **Figure 1**). This difference remained significant even after adjusting for potential confounding (coef=0.07 [95%CI= 0.007-0.13], p=0.028). Distribution of LS7 metrics varied between groups **(Figures 2a and 2b).** PLWH were more likely to have ideal BMI (aOR=3.27 [95%CI= 2.41-4.45], p< 0.001), ideal blood pressure (aOR=3.35 [2.56-4.37], p< 0.001) and ideal total cholesterol (aOR=1.67 [1.24-2.24], p= 0.001). By contrast, PLWH were less likely to have ideal smoking (aOR=0.40 [0.26-0.59], p< 0.001), ideal blood glucose (aOR=0.39 [0.30-0.51], p< 0.001).

**Figure:**
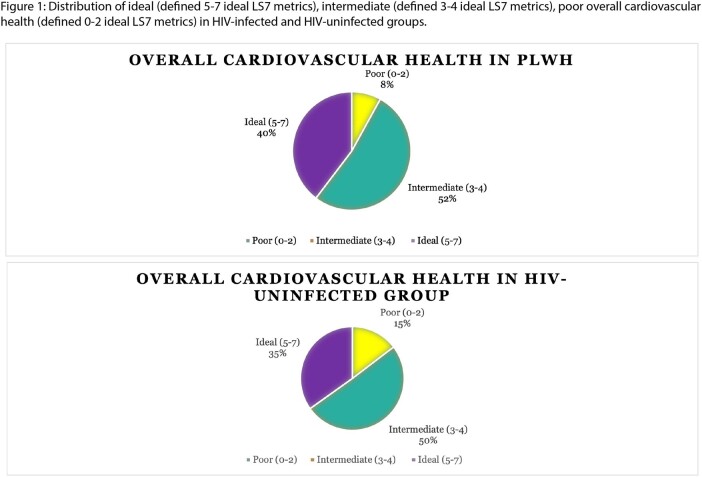

**Figure:**
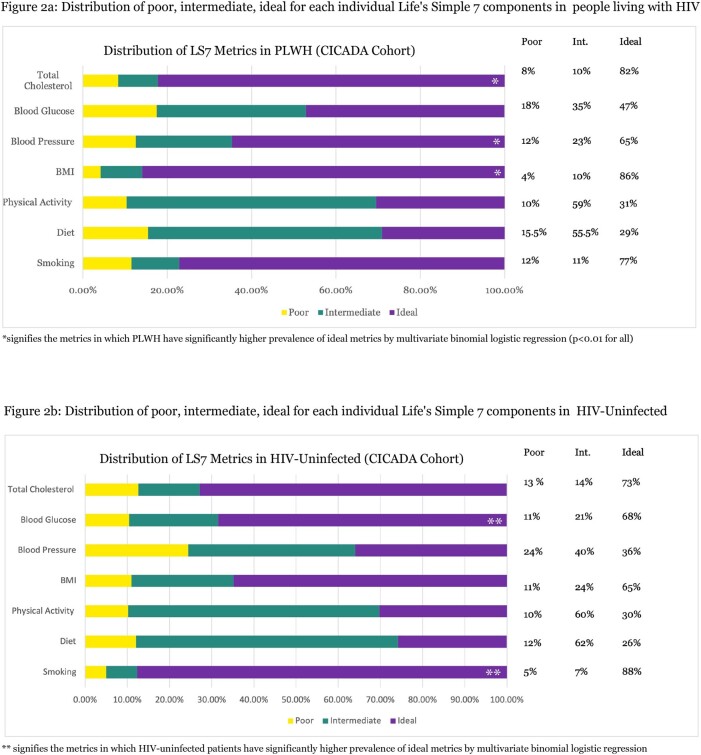

**Conclusion:**

Early HIV influences several key determinants of cardiovascular health encompassed in *Life's Simple 7* score. Our findings indicate favorable cardiovascular health in newly diagnosed PLWH, driven by lower rates of metabolic risk factors. However, such patients would still benefit from primary prevention efforts prioritizing smoking cessation, diabetes screening to mitigate remaining CVD risk.

**Disclosures:**

**All Authors**: No reported disclosures

